# A Neglected Detail in Sanitation

**Published:** 1914-01

**Authors:** 


### A Neglected Detail in Sanitation.

Imagine the contents of a privy vault scattered along highways, in masses too small to attract attention except occasionally.

Imagine faecal matter containing infectious organisms originating in a locality in which a certain disease is more or less endemic or epidemic, carried into a community in which that disease is rare or unknown, perhaps a thousand miles distant. The first assumption, by the law of chance, implies accidental contamination of shoes, and distribution of germs in dust. The second implies special danger on account of the general susceptibility to disease in peoples not immunized to any degree by previous experience with that disease.

We have used the word "Imagine" but we are not dealing with an imaginary case. The assumptions mentioned are being carried out continuously on a large scale by the railroads of the country. We do not care to take the trouble of entering into statistics, it ought to suffice to point out that every average railroad car, half full of passengers, represents, time for time, the equivalent of five or six average privy vaults. Everyone knows how the closets of these cars are emptied. If they were emptied in large masses, after a few days action of saprophytes, the abuse would not be tolerated except in a region very sparsely populated and lacking in sanitary conceptions and sanitary officials. But, the very fact that the evacuations are scattered so as to be inconspicuous and that pathogenic organisms have not been subjected to the action of saprophytes, renders the danger all the greater.

Of course, the great majority of travelers are healthy, from the sanitary standpoint but, on the whole, the traveling public is more liable to include persons in the incubation stage of disease, hurrying home, or those advanced in tuberculosis or other disease, seeking more salubrious climate, than the average ambulant population. Immigrants, returning soldiers, and long distance travelers generally are more apt to be infested with rare intestinal parasites than the ordinary population and it is a truism that the transfer of infections of a more or less local origin must occur mainly by travel.

It may be objected that railroad tracks are not highways, except for those in the cars and not especially liable to contamination from the closets, beyond the inevitable danger of any public closet. Theoretically this is true but, aside from the quite considerable number of employees working within and walking along the railroad reservations, the facts are that railroads are used to a considerable degree as foot paths, that they cross ordinary highways frequently, that closets cannot be closed in transit through small towns and that more or less contamination of large stations is liable to occur.

The chance of any particular evacuation being infected and being deposited so as to transfer infection if present, is very small. At a guess we should say that this chance is less than

1 : 1,000 but more than 1 : 1,000,000. But this chance is multiplied by many millions in the course of a year.

The remedy is obvious, practicable and not of prohibitive expense. Every traveling closet should have a receptacle attached and this should be disinfected and emptied at convenient times and places, subject to the same general principles as apply to the sanitary discharge of sewage otherwise.

